# The Effects of in-Season Repeated Sprint Training Compared to Regular Soccer Training

**DOI:** 10.1515/hukin-2015-0126

**Published:** 2015-12-30

**Authors:** Eirik Solberg Nedrehagen, Atle Hole Saeterbakken

**Affiliations:** 1 Department of Teacher Education and Sports of Sogn and Fjordane University College, Sogndal, Norway

**Keywords:** high intensity running, intermittent recovery test, Yo-Yo IR-1, soccer training, performance

## Abstract

The aim of this study was to compare the effects of repeated sprints (RSA) training and regular soccer training on Yo-Yo IR-1 and RSA performance (6 x 40 m shuttle sprints). Thirteen semi-professional female soccer players and nine amateur male soccer players were randomised into a repeated sprint group (RSG; n = 12) or a regular soccer training group (STG; n = 10). The RSG soccer players executed 3–4 sets of 4–6 repeated sprints (30 m with 180° directional changes) weekly during the last eight weeks of the in-season. In parallel, the STG soccer players performed low- to moderate intensity soccer training in form of technical or tactical skills. The RSG showed 15% improvement in Yo-Yo IR-1 (p = 0.04; ES = 1.83) and their mean RSA times were reduced by 1.5% (p = 0.02; ES = 0.89). No significant changes were found for the STG (Yo-Yo IR-1, p = 0.13; RSA, p = 0.49). Comparing the groups, greater improvements were observed in Yo-Yo IR-1 for the RSG (p = 0.02; ES = 1.15), but not for the RSA (p = 0.23; ES = −0.33). Similar training volumes and intensities (% of HFmax) were observed between the groups (p = 0.22 and p = 0.79). In conclusion, a weekly RSA session integrated into a regular soccer regime improved in-season RSA and Yo-Yo IR-1 performance compared to regular soccer training.

## Introduction

During soccer matches, periodic bouts of repeated high intensity runs (HIR) and sprints occur causing temporary fatigue and reduced performance, and may be crucial in the final minutes of soccer matches (Bradley et al., 2009; Rampinini et al., 2009b; Spencer et al., 2005). For example, reductions in HIR (>14,4 km/h-1) and sprints (>25 km/h-1) have been observed in the second half of soccer matches compared to the first half as well as during the final 15 minutes of match play (Bradley et al., 2009; Mohr et al., 2005). It is plausible that this reduction is related to reduced technical performance in the second half of soccer matches compared to the first half (Rampinini et al., 2009a). Therefore, gaining knowledge about effective interventions to reduce temporary fatigue and fatigue at the end of soccer matches would be of great value.

Basic physiological variables (i.e. maximal oxygen uptake, anaerobic threshold and maximal running speed) are widely used to improve physical soccer performance. Lately, repeated HIR and repeated sprint ability (RSA) tests have been constructed, validated and correlated with specific training physiology in soccer and training induced effects (Krustrup et al., 2003; Rampinini et al., 2007). In addition, it has been demonstrated that the ability to repeat HIR and repeated sprints (RSA) in tests can distinguish professional soccer players from amateurs, as well as teams of different standards at the same competitive level (Ingebrigtsen et al., 2014; Mohr et al., 2003; Rampinini et al., 2009b).

Several studies have compared RSA training regimes with different intervention programs (i.e. explosive strength training, small field soccer and technical drills) and demonstrated that RSA is superior in improving acceleration (Buchheit et al., 2010; Mujika et al., 2009). Other studies have demonstrated that RSA training is superior to aerobic intervals (4×4 min, 90–95% of HRmax) in improving HIR (Yo-Yo IR-1) and RSA test performance (Bravo et al., 2008). The isolated effect of RSA training is often difficult to determine, as intervention programs often combine RSA training with agility training and/or strength training (Shalfawi et al., 2013).

Pre-season RSA training twice a week for 6–10 weeks has been shown to improve sprint performance (acceleration and sprint time), RSA (10 x 40 m) and HIR (Yo-Yo IR-1 and IR-2) (Ingebrigtsen et al., 2013; Shalfawi et al., 2012; Tønnessen et al., 2011; Wong et al., 2010). However, those studies did not match the training volume between the intervention and control groups, which could explain the positive effects of RSA training. In addition, Gunnarsson and colleagues (2012) did not include a control group, but both demonstrated improved 40 m sprint and HIR (Yo-Yo IR-1 and IR-2) performances after a weekly in-season RSA session.

To the best of our knowledge, no previous studies had matched total training volume and intensity between intervention groups. Furthermore, no study had compared the effects of RSA with regular soccer training using two intervention groups. Therefore, the aim of this study was to compare the in-season effects of weekly repeated sprint training with regular soccer training controlling for training volume and intensity between the groups. It was hypothesized that 3–4 series of 4–6 repeated shuttle sprints for eight weeks would improve in-season Yo-Yo IR-1 (HIR) and RSA performances for the repeated sprint group, but not for the regular soccer training group.

## Material and Methods

### Participants

Forty senior soccer players from one amateur team playing at the local level (males, n = 20) and one semi-professional team (females, n = 20) playing at the national level were recruited as participants (Table 1). Thirteen females and nine males volunteered and fulfilled the inclusion criteria as licensed senior players (≥16 years). The subjects completed pre- and post-tests and participated in a minimum of seven out of eight training sessions (>80%) without limitations to maximal intensity in the form of injury or pain. During the intervention period, two participants were excluded from each group because of injury. Goalkeepers were not included in the research. The pre-test results were not significantly different between the RSG (n = 14, female: 8, male: 6) and the STG (n = 12, female: 7, male: 5) for any given variable (Table 1).

Prior to participation, all players were informed about the study and signed a written consent form. The research procedures were approved by the local ethics committee and conformed with the Declaration of Helsinki and the ethical guidelines of the Sogn og Fjordane University Colleges.

### Procedures

The study design was repeated measures within and between groups. Stratified randomisation was used to allocate the included participants based on sex and the competitive level to either the repeated sprint group (RSG) or the soccer specific training group (STG). RSG training was implemented weekly in the last eight weeks of the in-season. Each session consisted of 4–6 repetitions of 20+10 m shuttle runs repeated in 3–4 series in a progressive training program. Parallel to RSG training, the STG performed specific soccer training in the form of technical and tactical skills at low to moderate intensity controlled by heart rate monitoring. The implemented repeated sprint training constituted 20–30 min as the single factor discriminating it from regular soccer training. Before and after the intervention, all participants were tested by means of the YoYo IR-1 test and repeated sprint ability (see testing procedures).

### Testing procedures

The participants were instructed to avoid strenuous exercise and all training 48 and 24 hours prior to the pre- and post-tests, respectively. The Yo-Yo IR-1 was conducted a minimum of 24 h and a maximum of 72 h before the repeated shuttle sprint test. All tests were conducted at the same time of day on the same artificial indoor soccer pitch with participants using soccer shoes with knobs.

#### Yo-Yo IR-1

The participants were tested in groups of four using heart rate monitors (Polar 300 RX, Polar Electro, Kempele, Finland) with coded belts. The maximal heart rate achieved during the Yo-Yo test was used as the reference value for the maximum heart rate. Before receiving oral information from the test leader and test-CD (The Yo-Yo tests, www.bangsbosport.com), the participants underwent 10 min of a general warm up individually. The YoYo IR-1 consisted of repeated 2×20 m shuttle runs with progressively increasing speed adjusted by audio signals from the test-CD. Between the shuttles, the participants underwent 10 s of active recovery consisting of 2×5 m of jogging or walking (Krustrup et al., 2003). As an absolute criterion, the participants had to stand completely still at the starting point before doing the next shuttle run. The test was terminated the second time the participants failed to cross the finish line before the signal. The last approved completed shuttle and the maximum heart rate were manually noted by the test leader for further analysis. A blinded test assistant and the test leader ensured that the participants were running correctly at each end of the shuttle. For a more detailed protocol description, see Krustrup and colleagues (2003).

### Repeated sprint ability

Prior to repeated sprint testing, the participants underwent 15 min of a general warm-up including three submaximal shuttle runs in the test area. The test consisted of six maximal 2×20 m shuttle runs with 20 s of passive recovery between the runs. The subjects executed a single maximal pre-trial used as a reference for maximal intensity 5 min before the experimental test. If the first of the six repeated sprints was ≥ 2,5 % slower than the pre-trial time, the test was terminated, and a new trial was conducted 5 min later. As an absolute criterion, the participants had to touch the 20 m line with at least one foot before turning 180 degrees. The test leader controlled the photocell system (Musclelab 4010, Ergotest Innovation A/S, Porsgrunn, Norway) and noted the results manually while a test assistant observed the participants’ turns. Time started when the participants crossed the first photocell after a flying start from a marked line 30 cm ahead (Duthie et al., 2006). Between the repeated sprint runs, the test leader informed the subjects when 10 s passed and then counted down from 5 s until the start of the next sprint. The mean sprint time for all six runs was used for further analysis (Impellizzeri et al., 2008). A detailed protocol is described elsewhere (Rampinini et al., 2007).

### The training intervention

RSG training was implemented weekly in the most intense session the last eight weeks of the in-season. After warm-up procedures, each session consisted of 4–6 repetitions of 20+10 m shuttle runs repeated in 3–4 series in a progressive training program (Table 2). A five minutes recovery period was conducted between the series with 30 s between repetitions at a 1:5 ratio (Abt et al., 2011; Little et al., 2007). To calculate the recovery time between the repetitions, a photocell registered the mean 30 m split-time during the pre-test. The mean sprint time was 5.92 (±0.32) s, and with a 1:5 ratio, the recovery time between repetitions was ~30 s. The recovery periods were passive to ensure maximal restitution between the repetitions and series. An experienced elite physical soccer coach supervised all repeated sprint sessions to ensure that they were executed at a high quality level. Parallel to RSG training, the STG performed specific soccer training in the form of technical and tactical skills at low to moderate intensity controlled by heart rate monitoring. The implemented repeated sprint training constituted 20–30 min as the single factor discriminating it from regular soccer training. All individual and team organized training was systematically registered by participants and their teams’ head coaches using an online tool specifically constructed for soccer teams (www.treningsdagbok.org). These data were supplemented by heart rate monitoring (Polar 300RX with coded belts, Polar Electro, Kempele, Finland) in intervention-sessions, which allowed for control of intensity and training volume between the groups. Total training time (i.e., individual and team organized training, including match time) and a mean heart rate during the intervention sessions were registered for further analysis.

### Statistical Analysis

All data are presented as the means ± standard deviations (SD). Unpaired t-tests were used to detect group differences at baseline. We used a two-way analysis of variance (ANOVA) to compare the Yo-Yo IR-1 and repeated sprint results of the two training groups (RSG and STG) and to compare the participants’ testing times (pre- and post-tests) with their Yo-Yo IR-1 and RSA-test results. When an interaction was detected, Bonferroni corrections were used to correct for the differences. The meaningfulness of the effects are presented by means of Cohens’d effect sizes (ES); with trivial (<0.20), small (0.20–0.50) medium (0.50–0.79) or large (>0.80). The level of significance was set as *p*>0.05.

## Results

### Repeated sprint ability

A significant group x time interaction was detected (F = 7.39, *p* = 0.02). The comparison of the pre- and post-test results in mean sprint time results after post hoc correction demonstrated a decrease of 1.41% in the RSG (*p* = 0.02, ES = 0.89), but no decrease for the STG (*p* = 0.49, ES = 0.26). No significant differences were observed in the pre-test (*p* = 1.00) or post-test results between the groups (*p* = 0.23, ES = 0.33; Table 3).

#### Yo-Yo IR-1

A significant group x time interaction was observed (F = 21.72, *p* < 0.01). The comparison of pre- and post-test results after post hoc corrections demonstrated an increase in total distance of 15,3% in the RSG (*p* < 0.05, ES = 1.83), but an insignificant reduction of 7.7% in the STG (*p* = 0.13, ES = −0.56). Similar total distances were observed in the pre-tests between the groups (*p* = 0.72), but the RSG had a significant greater total distance in the post-test (*p* = 0.02, ES = 1.15; Table 3).

### Training load

The intervention training was 7% of the RSG and 8% of the STG weekly training time (25 min weekly). No significant differences between the groups were detected in weekly training time (RSG: 6.34 ± 2.42 hours vs STG: 5.23 ± 1.67 hours, *p* = 0.22) during the intervention period or in training intensity during the intervention sessions (RSG: 75.6% ± 5.2 HF_max_ vs STG: 74.3% ± 8.4 HF_max_, *p* = 0.79; Table 3).

## Discussion

The present study demonstrated an improvement in Yo-Yo IR-1 and RSA performance after weekly repeated sprint training, but not after weekly regular soccer training. The RSG demonstrated greater improvements in Yo-Yo IR-1 performance than the STG, while no group differences were observed in RSA performance. Similar training volume and intensity were observed between the groups during the intervention period.

Consistent with our hypothesis, repeated sprint training, but not regular soccer training, improved RSA performance. Greater training specificity effects of repeated sprint training on RSA test performance probably accounted for the improvements in RSA performance in the RSG, especially the improvements in 180 degree turns, lactate buffer capacity, anaerobic capacity and ability to accelerate/decelerate (Buchheit et al., 2012). Buchheit and colleagues (2012) demonstrated that the effects of repeated sprints with directional changes were angle dependent. However, specific learning effects of the RSA protocol in the RSG may have also contributed to the RSA performance improvements. Furthermore, a small but important performance change could have been overlooked, as a four week reliability study concluded that the mean RSA sprint time was only reliable for detecting large training induced changes (Impellizzeri et al., 2008). A limitation of the study is that no familiarization sessions were conducted before the RSA performance pre-test to measure the short-term learning effect. However, Impellizzeri and colleagues (2008) revealed a standard error of 0,8% among professional soccer players.

Importantly, similar training volume and training intensity between the groups were observed. The short period of increased training intensity of the weekly RSA training compared to traditional soccer training did not significantly increase the mean training intensity for the RSG compared to the STG. Previous studies examining RSA training effects have involved extra training sessions compared to the control group, which may have influenced the study results (Shalfawi et al., 2012; Shalfawi et al., 2013; Tønnessen et al., 2011; Wong et al., 2010).

Our RSA results are supported by several studies (Bravo et al., 2008; Buchheit et al., 2010; McGawley et al., 2013), but are in contrast to reports of no effect within group (Ingebrigtsen et al., 2013; Shalfawi et al., 2013) and reports of effects between groups (Shalfawi et al., 2012; Tønnessen et al., 2011). Importantly, the results of several previous studies are not directly comparable to the present study, as the training volumes between the groups were not matched, different competitive levels were examined, there was no control group and different training approaches were conducted. However, an in-season study by Bravo et al. (2008) used a RSA testing protocol identical to ours. They conducted training twice per week over seven weeks and demonstrated a 2.1% decrease in mean repeated sprint time, which supports our findings.

Consistent with our hypothesis, RSA training improved Yo-Yo IR-1 performance and resulted in greater improvements than regular soccer training, possibly due to muscular and aerobic adaptations caused by RSA training (Bravo et al., 2008; Gunnarsson et al., 2012). Furthermore, it is important to note that both tests (RSA and Yo-Yo IR-1) involve turnings. Therefore, it is possible that adaptations in the ability to turn as a result of RSA training could have skewed the Yo-Yo IR-1 results in the positive direction (Buchheit et al., 2012). No familiarization trial was executed before the YoYo IR-1 pre-test. However, Krustrup and colleagues (2003) reported that the first Yo-Yo IR-1 test provided reliable results among adult men.

The results of the present study are supported by those of Bravo and colleagues (2008) who also analyzed Yo-Yo IR-1 performance in an in-season study. They observed a ~28 % increase in Yo-Yo IR-1 performance after implementing RSA training over seven weeks. In contrast to the present study, Bravo and colleagues (2008) implemented 180 degree directional changes every 10th m of the 40 m sprints in the first three training weeks, used less recovery time, performed RSA training twice a week and used junior soccer players as participants. All of those factors may have resulted in the greater improvement in Yo-Yo IR-1 performance that was observed in that study compared to the present one.

The RSG had greater improvements in repeated high intensity running (HIR) measured by the Yo-Yo IR-1 than the STG, and this result is supported by those of Wong and colleagues (2010). They compared regular soccer training with two high intensity interval sessions (16 intervals of 15 s of 120% of aerobic running speed) and heavy strength training (4–6RM) weekly over eight weeks in professional soccer players. The combined strength and repeated high intensity running group showed 8% greater improvements in Yo-Yo IR-1 performance than the traditional soccer training groups. This difference is substantially smaller than our findings (8% vs. 30%). This contrast may demonstrate that a specific training stimulus is superior to general training qualities in terms of improving HIR.

The present study was limited by the use of self-reported results of the total training volume in the analyses and the use of mean training intensity (% of the maximum heart rate) and not the methods that specifically measured intermittent running. Therefore, future studies should use more accurate methods for monitoring training loads (i.e. tracking systems as they are suitable for categorizing intermittent workload). Although the RSG showed improved HIR and RSA performance, the effects of RSA training on in-game physical performance is still uncertain and should be investigated in the future using tracking systems. Lastly, dose-response and sprint/recovery ratios should be tested in a range of competitive levels.

In conclusion, this study demonstrated that weekly RSA training improved in-season Yo-Yo IR-1 and RSA performance compared to regular soccer training. The Yo-Yo IR-1 improvement in the RSG was greater than in the STG. Therefore, we recommend integrating 3–4 series of 4–6 sprints weekly into regular soccer sessions to improve HIR and RSA performance in-season in amateur and semi-professional players.

## Figures and Tables

**Table 1:** Basic characteristics of subjects in the repeated sprint group (RSG) and the soccer training group (STG) (mean ± SD)

	Female semi-professionals (n= 13)	Male amateurs (n=9)	RSG (n=12)	STG (n=10)
Age (years)	19.9±2.5	22.0±2.7	20.3±3.0	21.8±2.6
Body height (cm)	168.1±6.0	180.7±3.4	67.6±13.9	68.6±9.9
Body mass (kg)	61.5±9.1	77.6±8.9	173.3±9.2	173.1±7.0
Years as senior players	4.9±2.5	6.5±3.0		

**Table 2:** The RSG training program

Week	Series	Repetitions	Total distance (m)	Length (m)	Pause/rep. (s)	Pause/series (min)
1	3	4	360	30	30	5
2	3	5	450	30	30	5
3	3	6	540	30	30	5.
4	4	4	480	30	30	5
5	4	5	600	30	30	5
6	4	6	720	30	30	5
7	3	6	540	30	30	5
8	3	5	450	30	30	5

**Table 3:** Mean results (± SD) for the Yo-Yo IR-1 and 6×40 m shuttle sprint, mean total training time included match play in the intervention period and a mean heart rate in intervention sessions between groups

	RSG		STG	

	Pre	Post	Pre	Post
Yo-Yo IR-1 (m.)	1455±188	1677±308*†	1409±336	1291±365
RSA (s)	7.79±0.37	7.68±0.41*	7.79±0.50	7.83±0.49
Total training time (hours)	46.8±19.4		41.9±13.4	
Heart-rate intervention sessions (% HF max)	75.6±5.2		74.3±8.4	

* Significant difference within group

† Significant difference between groups
